# Seminal plasma inhibits *Chlamydia trachomatis* infection in vitro, and may have consequences on mucosal immunity

**DOI:** 10.1038/s41598-024-71499-9

**Published:** 2024-09-09

**Authors:** Louis Reot, Cindy Adapen, Claude Cannou, Natalia Nunez, Sabrine Lakoum, Camille Pimienta, Laetitia Lacroix, Olivier Binois, Nelly Frydman, Marie-Thérèse Nugeyre, Roger Le Grand, Elisabeth Menu

**Affiliations:** 1 Center for Immunology of Viral, Auto-Immune, Hematological and Bacterial Diseases [IMVA-HB/Infectious Disease Models and Innovative Therapies (IDMIT)], Commissariat à l’Energie Atomique et aux Energies Alternatives (CEA), Inserm, Université Paris-Saclay, Fontenay-aux-Roses, France; 2 Mucosal Immunity and Sexually Transmitted Infection Control (MISTIC) Group, Department of Virology, Institut Pasteur, Université Paris Cité, Paris, France; 3 Life&Soft, Fontenay-aux-Roses, France; 4 Service de Biologie de la Reproduction CECOS, Assistance Publique Hôpitaux de Paris, Hôpital Antoine Béclère, Université Paris-Saclay, Clamart, France

**Keywords:** STI, *C. trachomatis*, Seminal plasma, Inflammation, Female mucosa, Neutrophil, Immunology, Infectious diseases, Inflammation, Innate immune cells, Mucosal immunology

## Abstract

Seminal plasma (SP) is the main vector of *C. trachomatis* (CT) during heterosexual transmission from male to female. It has immunomodulatory properties and impacts the susceptibility to HIV-1 infection, but its role has not been explored during CT infection. In the female reproductive tract (FRT), CT infection induces cytokine production and neutrophil recruitment. The role of neutrophils during CT infection is partially described, they could be at the origin of the pathology observed during CT infection. During this study, we developed an experimental in vitro model to characterize the impact of CT infection and SP on endocervical epithelial cell immune response in the FRT. We also studied the impact of the epithelial cell response on neutrophil phenotype and functions. We showed that the production by epithelial cells of pro-inflammatory cytokines increased during CT infection. Moreover, the pool of SP as well as individuals SP inhibited CT infection in a dose-dependent manner. The pool of SP inhibited cytokine production in a dose-dependent manner. The pool of SP altered gene expression profiles of infected cells. The culture supernatants of cells infected or not with CT, in presence or not of the pool of SP, had an impact on neutrophil phenotype and functions: they affected markers of neutrophil maturation, activation and adhesion capacity, as well as the survival, ROS production and phagocytosis ability. This study proposes a novel approach to study the impact of the environment on the phenotype and functions of neutrophils in the FRT. It highlights the impact of the factors of the FRT environment, in particular SP and CT infection, on the mucosal inflammation and the need to take into account the SP component while studying sexually transmitted infections during heterosexual transmission from male to female.

## Introduction

Sexually transmitted infections (STI) represent a major public health issue, with a growing incidence of several STI, despite the existence of different treatments. Over the last years, *Chlamydia trachomatis* (CT) infection cases increased worldwide, especially in young people^1,2^. CT is a Gram-negative bacterium that infects preferentially epithelial cells. Serovars D to K are responsible for urogenital infections. Most of the cases of CT infection are asymptomatic, and the infected population represent a reservoir for STI spreading, so there is a real need for prevention methods. One of the key issues to develop new preventive strategies is to understand more deeply the role of the local environment during CT acquisition.

In the female reproductive tract (FRT), CT infection results in a pro-inflammatory cytokine and chemokine response that leads to the recruitment of innate, and later adaptive immune cells, allowing the control of the infection^3–5^. Persistence of the pathogen results in chronic inflammation and can lead to collateral genital tract tissue damage^6,7^. Polymorphonuclear leucocytes (PMN) from the blood are the first immune cells recruited to the site of CT infection. Through various antimicrobial activities (degranulation, phagocytosis, reactive oxygen species (ROS) and neutrophil extracellular traps (NET) production …), they allow pathogen clearance. However, the role of neutrophils during CT infection is not fully understood. They could induce the immune pathology during CT infection^8^: in an in vitro study, CT enhanced neutrophil responses, characterized by a recruitment of neutrophils to the site of infection, their activation and prolonged survival^9^. However, in other experimental settings, neutrophil responses are counteracted, and neutrophils are paralyzed by CT^10^. Moreover, in in vivo mouse models, neutrophils can contribute to antibody-mediated protection to CT infection^11^, or on the contrary, drive CT associated pathology, without reducing the bacterial burden^8^. These studies highlight the need to better characterize the role of those cells during CT infection.

During heterosexual transmission from male to female, the main vector of STI pathogens in the FRT is the seminal plasma (SP)^12,13^. Very little is known about the role of seminal plasma during CT induced pathology. SP, the acellular fraction of the semen, is composed of various proteins, including pro and anti-inflammatory cytokines^14^. It is known to induce an inflammatory reaction in the FRT, leading to cytokine production and the recruitment of neutrophils from the blood^15,16^. The inflammation induced by SP exposure in the FRT has been linked to the regulation of infectious diseases, notably modifying the risk for HIV-1 acquisition^17,18^. SP also has strong antimicrobial properties conferred by proteolytic cleavage of semenogelins (the main protein of human semen coagulum), displaying bactericidal activity against various Gram-positive and Gram-negative bacteria^19^. However, even if SP has been shown to modulate STI susceptibility and inflammation at the level of the FRT^20^, the impact of SP is poorly investigated during CT infection.

In this study, we aimed at characterizing the impact of CT infection in presence or in absence of SP on epithelial cell response at the level of the FRT mucosa. We also investigated the impact of the epithelial cell response on the phenotype and functions of neutrophils. Using an in vitro model, we found that SP inhibited CT infection and modified the inflammation induced by the infection, in particular the cytokine profile of CT infected epithelial cells. We also show that the local environment could have an impact on local neutrophil phenotype and functions.

## Material and methods

### Seminal plasma collection

Human seminal plasmas were obtained from the “*Centre d’étude et de conservation des œufs et du sperme humains*” (CECOS) at Antoine Béclère Hospital. Semen was collected from patients that were involved in in vitro fertilization protocol. All methods to recruit participants and collect biological samples were carried out in accordance with relevant guidelines and regulations. All participants gave their written informed consent for the use for medical research purposes of the samples. They were informed of the study and gave no objection for the use of their samples and data for the study.

The study was defined as a non-interventional study by French Ethical Committee ‘Comité de Protection des personnes’ (no. 2014/42NICB). The study complies with the methodology reference MR-004 set out by the French data protection authority (‘Commission Nationale de l’Informatique et des Libertés’ CNIL) for which Institut Pasteur Paris made a statement compliance with the CNIL (no. 2214728v0). The study was registered on the public directory of the health data hub HDH (No. F20220617111005).

All patients had a negative serology for HBV, HCV, HIV and Syphilis. A spermaculture was systematically carried to detect any bacterial infections, semen was also tested for Chlamydia and Mycoplasma by PCR. SP were obtained after semen centrifugation on gradient density.

SP from about 100 patients were pooled, aliquoted and characterized for cytokine concentration. Thirty-two individual SP were also stored.

### Cell line culture

A2EN is a human endocervical epithelial cell line, generated in the laboratory of Dr A. Quayle from primary epithelial cells isolated from endocervical explant, and immortalized with human papilloma virus E6 and E7^21^. A2EN cells (Kerafast, MA, USA) were grown in phenol red-free serum-free medium (EpiLife; Cascade Biologics) with an Epilife Defined Growth Supplement (EDGS; Gibco), 1% Penicillin/Streptomycin (PS) and 0.004 M of CaCl_2_ (Sigma Aldrich). The cells were grown at 37 °C with 5% CO_2_. High molecular weight (HMW) poly(I:C) (100 µg/mL) was used as a positive control to stimulate A2EN cell cytokine production.

### *C. trachomatis* infection

*Chlamydia trachomatis* serovar D (D/UW3/Cx) was obtained from Statens Serum Institut (Copenhagen, Dr Follmann laboratory). A2EN cells were seeded in EpiLife medium with EDGS, CaCl_2_ and PS in 24 well plate at 5 × 10^4^ cells/well. After 96 h, confluent A2EN cells were washed, and fresh Epilife medium without PS containing, or not, various dilutions of SP (1/10, 1/50, 1/100 or 1/500) was added. The cells were immediately infected with CT svD at different multiplicity of infection (MOI) (12, 25 or 50) by centrifugation (700*g*) for 1 h. Then, culture medium was removed and DMEM with 10% FCS was added. The cells were incubated for 24 h at 37 °C with 5% CO_2_. After 24 h, pH measurement (Fisherbrand pH Indicator, 6.4–8) and viability test (CellTiter) were performed. Supernatants were collected and frozen at −80 °C after 0.2 μm filtration. For the determination of the percentage of infection, cells were fixed and stained using a rabbit anti-chlamydia heat shock protein 60 (Hsp60) antibody (provided by A. Subtil, Institut Pasteur, France) diluted at 1:2000 in PBS + 1% BSA for 1 h. Then, cells were treated with anti IgG Rabbit-AF488 (1/500), DAPI and Phalloidin for 1 h. Inclusions were quantified in infected cells using an inverted microscope (Zeiss Axiovert 25). The percentage of infection was determined by dividing the number of inclusions containing cells by the total number of cells.

### RNA extraction and sequencing

At different timepoints, cells were lysed and lysates stored at −80 °C for RNA extraction. RNA was extracted using NucleoSpin RNA XS kit (Macherey Nagel) according to manufacturer instructions. RNA was quantified using QuBit RNA HS kit (ThermoFisher), and a quality check was performed on the Agilent TapeStation system. A total of 1000 ng of RNA per sample was denatured at 65 °C and retrotranscribed by a strand-switching technique using Maxima H Minus Reverse Transcriptase (ThermoFisher, USA) to synthesize a double stranded cDNA. PCR, barcode, and adapter attachment were performed according to cDNA-PCR Sequencing Kit (SQL-PCB109, Oxford Nanopore Technologies, Oxford, UK). Samples were quantified using QuBit dsDNA HS (ThermoFisher, USA) kit before loading on R9.4.1 Flow cells using the GridION instrument (Minknow version 21.11.7).

### Transcriptome analysis

Sequence reads were converted into FASTQ files. Reads under 300 bp or with a quality score under 9 were discarded. The remaining reads were aligned on the human GRCh38.p13 and *C. trachomatis* D/UW-3/CX strain transcriptome of reference (GeneBank assembly accession numbers GCA_000001405 and GCA_000008725 respectively) using minimap2^22,23^ version 2.24. To quantify transcripts, the resulting alignments were given to Salmon version 1.8.0^24^. To explore differentially expressed genes, replicates count data were used on DESeq2 version 1.32.0^25^ in one single DEseq model. Gene set enrichment analysis with both upregulated and downregulated genes Log2FC > 1.5 or Log2FC < 1.5, respectively was performed using Enrichr, a web server enrichment analysis tool^26,27^, and BioPlanet 2019 database for cellular and signaling pathway analysis^28^.

### Cytokine and chemokine quantification

Pro- and anti-inflammatory cytokines were measured in the A2EN cell culture supernatants and in the seminal plasma by a 25plex assay for the detection of: IL-1β, IL-1RA, IL-2, IL-2R, IL-4, IL-5, IL-6, IL-7, IL-8, IL-10, IL-12 (p40/p70), IL-13, IL-15, IL-17, TNFα, IFNα, IFNγ, GM-CSF, CCL3, CCL4, CXCL10, CXCL9, CCL11, CCL5 and CCL2 (Human cytokine magnetic 25-plex panel; Life technologies).

TGF-β1, 2 and 3 were detected with another multiplex assay (MILLIPLEX MAP TGFß Magnetic Bead 3 Plex Kit; Merck Millipore).

### Neutrophil isolation, phenotype and functional analysis

Human neutrophils were isolated from human blood, obtained from blood donors at *Etablissement Francais du Sang* (EFS, France; C CPSL UNT-No. 13/EFS/101), using the EasySep Direct Human Neutrophil Isolation Kit according to the manufacturer’s instructions (StemCell Tech, Vancouver, BC, Canada). Each sorting resulted in more than 90% neutrophils, confirmed by phenotype analysis. 2.10^5^ neutrophils were incubated at 37 °C or at 4 °C (only for the phagocytosis assay) in a 96-well plate containing an equal volume of RPMI with 10% FCS and of A2EN cell supernatants or DMEM with 10% FCS. Phenotype and functional analysis were performed at various time post-incubation.

Survival of the neutrophils was followed at 2 h, 6 h, 24 h and 48 h using an apoptosis detection kit with PE-Annexin V and 7-AAD (Biolegend), according to the manufacturer instructions. At 2 h post-incubation, neutrophils were tested for ROS production and phagocytosis capacity.

For the phagocytosis assay, pHrodo Red *E. coli* BioParticles Conjugate for phagocytosis (Invitrogen) were added to the neutrophils at a 1:1 ratio for 5 min, then cells were washed and fixed using 1% PFA until flow cytometry analysis.

For the ROS production assay, neutrophils were washed and incubated at 37 °C for 90 min in PBS containing 10 µM Luminol (Sigma-Aldrich). PMA (0.1 ng/µL, Sigma-Aldrich) was added 0 min or 50 min after the addition of the Luminol. Luminescence was followed every min using a multimode microplate reader, the Spark 10M (TECAN; Switzerland). The phenotype of the cells was analysed after 6 h of incubation, after staining of the neutrophils for 10 min at 4 °C with the antibodies listed in Table 1. A ZE5 flux cytometer was used (Biorad) with Everest (Biorad) and FlowJo (Tristar, USA) software packages for the flow cytometry analysis. A representative image of the gating strategy is illustrated in Supplementary Fig. 1.

**Table 1:** Antibody panel used to characterize neutrophil populations.

Antibody	Clone	Label	Laser-filter	Reference	Volume/test (µL)
Bluevid			355-450/50		
CD64	10.1	BUV737	355-740/35	564425	3
CD14	REA599	Vioblue	405-450/50	130-110-5224	2
CD45	REA1023	Viogreen	405-525/50	130-177-193	2
CD3	SP34.2	BV650	405-660/20	563916	2
CD123	7G3			563405	5
CD8a	RPAT8			563821	2
CD20	2H7			563780	2
CD62L	SK11	BV711	405-710/50	565040	2
CD11b	REA713	FITC	488-530/30	130-110-552	2
CD10	HI10a	PercP-Cy5,5	488-695/40	312216	5
CDw125	REA705	PE	561-585/15	130-710-544	2,5
PD-L1	29E.2A3	PE-Dazzle 594	561-610/20	329732	3
CD101	REA954	PE-Vio770	561-780/60	130-115-832	2
CD32a	IV.3	AF647	640-670/14	60012	0,6
HLA-DR	L234	AF700	640-730/45	307626	1
CD66	TET2	APC-vio770	640-780/60	130-101-132	2

### Statistical analysis

GraphPad prism software version 9 for windows (GraphPad Software, La Jolla California USA, https://www.graphpad.com) was used for graphical representations and area under the curve (AUC) calculation. Two-way ANOVA test with p values adjustment with Tukey’s test was used for statistical analysis.

## Results

### Seminal plasma composition

**Fig. 1:**
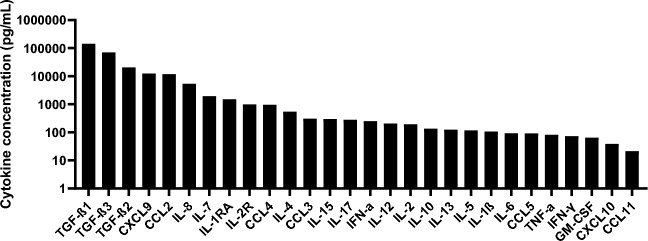
Cytokine characterization of the pool of SP. Seminal plasma from about 100 patients were pooled and the cytokine concentrations of the pool were quantified by Luminex. Figure was formulated using GraphPad Prism software.

The pool of SP was characterized in terms of cytokine/chemokine concentrations (Fig. 1). The pool of SP had a moderate inflammatory profile, with some pro-inflammatory cytokines expressed (IL-8, CCL2, CXCL9) but also anti-inflammatory cytokines (TGF-β, IL-1RA). As expected^29^, TGF-β1, -β3 and –β2, respectively, were the most prevalent cytokines in seminal plasma.

Since the presence of chlamydia-specific antibodies (from currently infected or previously exposed individuals) in seminal plasma would be a major confounder, anti-*C. trachomatis* IgG antibodies in seminal fluids (pool and individual) were measured using an indirect chemiluminescent immunoassay technique (CLIA; LIAISON Chlamydia trachomatis IgG "Diasorin"). All tested samples were negative (concentration < 5 Units/mL) (data not shown).

### Seminal plasma inhibited *Chlamydia trachomatis* infection of A2EN cells

The effect of the pool of SP was examined on A2EN cells infected with CT. Since pure SP could affect the viability of epithelial cells in vitro, we tested several dilutions of SP in PBS, starting with a tenfold dilution, to approximate the physiological situation in the FRT after intercourse^30^. No effect was detected on cell viability: the pH of A2EN supernatants and the metabolic activity of the cells were similar in all the conditions tested (data not shown). We then determined the percentage of CT infection in the various conditions by immunofluorescence.

**Fig. 2:**
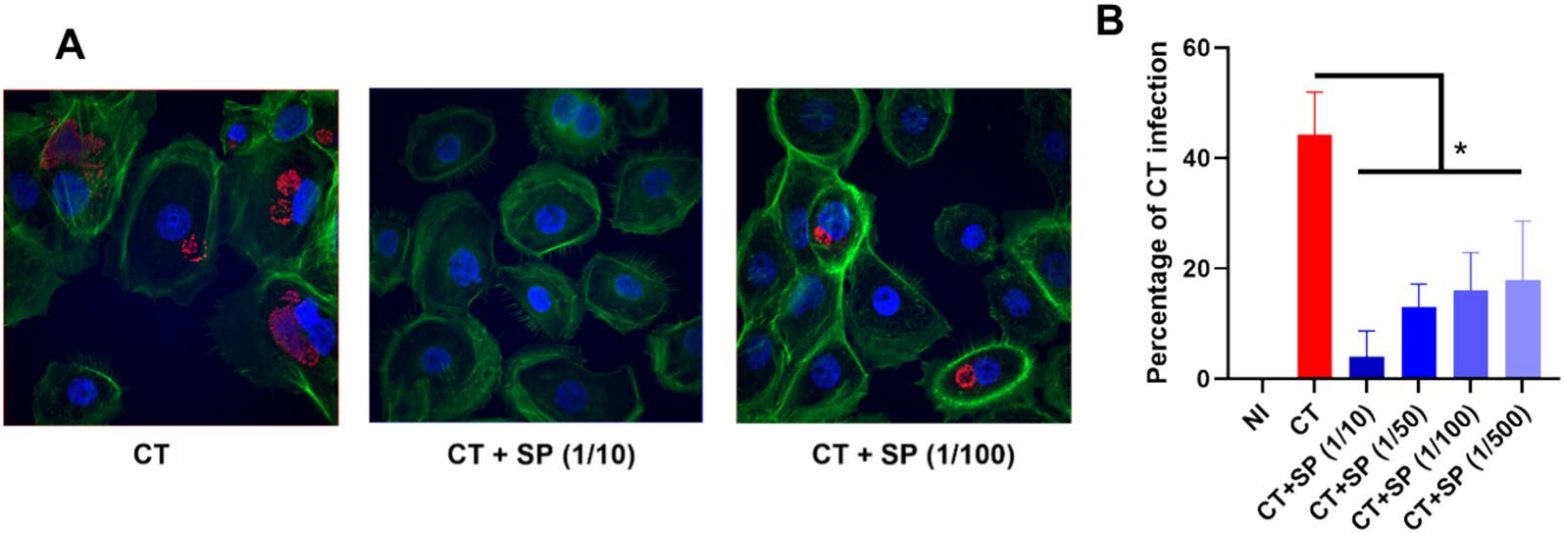
Impact of the pool of SP on CT infection of A2EN cells. Cells were infected or not with CT at a MOI of 12, in the presence or not of different dilutions of the pool of SP, for 24 h (n = 4). (**A**) The percentage of infection was determined by immunofluorescence. Actin filaments in the cytoplasm are in green (Phalloidin), the nucleus in blue (DAPI), and the CT inclusions in red (Hsp60 antibody). (**B**) Percentage of CT infection ([number of infected cells/total number of cells] × 100) at 24 hpi by quantification of the inclusion in A2EN infected cells. The asterisk indicates a significant difference between CT and CT + SP at all dilutions by T-test (*p ≤ 0.05). CT + SP (1/10) were also significantly different from other CT + SP dilutions by T-test (p ≤ 0.05). (**B**) Figure was formulated using GraphPad Prism software.

**Fig. 3:**
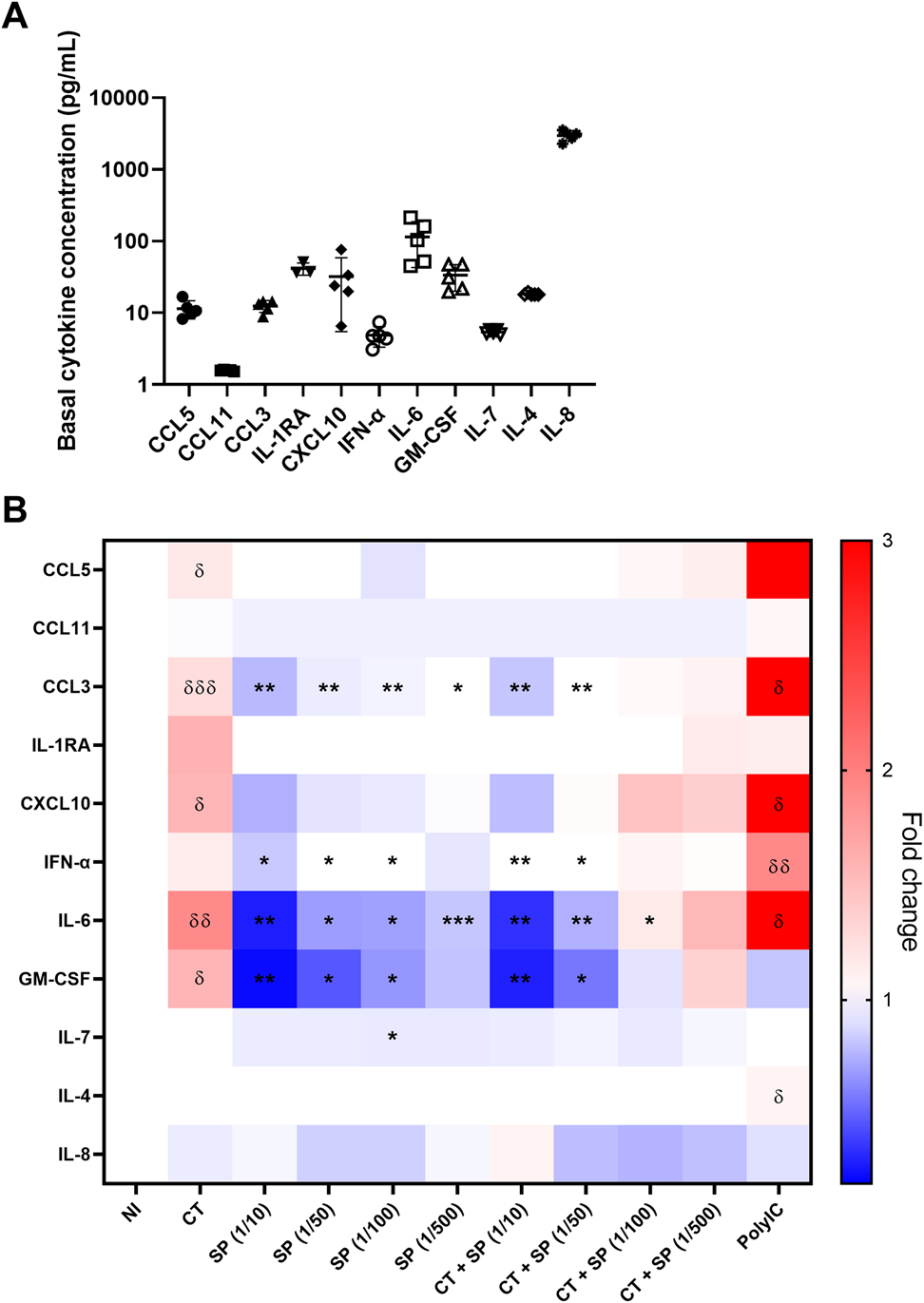
Impact of the pool of SP on cytokine expression by A2EN cells infected with CT. Cells were infected or not with CT at a MOI of 12 for 24 h, in the presence or not of different dilutions of the pool of SP (n = 5). Poly(I:C) stimulation was used as a positive control. The cytokines in the culture supernatants were quantified by Luminex. (**A**) Cytokine concentration in uninfected A2EN cells. Only detectable cytokines are shown. (**B**) Variation in cytokine expression after CT infection, with or without SP treatment, relative to non-infected (NI) condition. Increase in cytokine concentration is represented in red, decrease in blue. Asterisks indicate a significant difference between NI and CT or poly(I:C) by one sample t-test (δ p ≤ 0.05, δδ p ≤ 0.01, δδδ p ≤ 0.001) or between CT and SP or CT + SP by two-way ANOVA test (*p ≤ 0.05, **p ≤ 0.01, ***p ≤ 0.001). Figures were formulated using GraphPad Prism software.

A representative image is illustrated in Fig. 2A. We quantified the cells that had at least one inclusion and determined that the presence of SP drastically reduced the percentage of CT infected cells analyzed 24 h post-infection (Fig. 2B). This effect was dose dependent: the percentage of infection decreased with the SP concentration. The tenfold dilution of SP was the most potent to reduce the percentage of CT infection, with tenfold less infected cells compared to the condition without SP. No significant difference in the size of the inclusions was observed between the various experimental conditions (Supplementary Fig. 2). We then tested if this effect could be observed using different experimental conditions. The results represented in Supplementary Fig. 3 show that SP also inhibited CT infection at higher MOI or at 48 h post-infection. Uninfected A2EN cells produced various cytokines including CXCL10, IL-6, GM-CSF and a high concentration of IL-8 (Fig. 3A). Stimulation with poly(I:C), strongly increased A2EN cell production of several proinflammatory cytokines, including CCL5, CCL3, CXCL10 and IL-6 (Fig. 3B). CT infection induced an increase in inflammatory cytokines concentration (IL-6, GM-CSF and CXCL10), but lower than poly(I:C). In contrast, when cells were exposed to SP alone, a decrease in the concentrations of those same cytokines was observed in a dose dependent manner (Fig. 3B). When A2EN cells were exposed to CT in presence of SP, the cytokine profile was dependent on the dilution of SP: the effect of SP predominated for low dilutions, with a decrease in cytokine concentration. On the contrary, for high dilutions, the effect of CT predominated, with an increase in inflammatory cytokine concentration. In conclusion, SP inhibited CT infection and decreased the inflammation induced by CT.

**Fig. 4:**
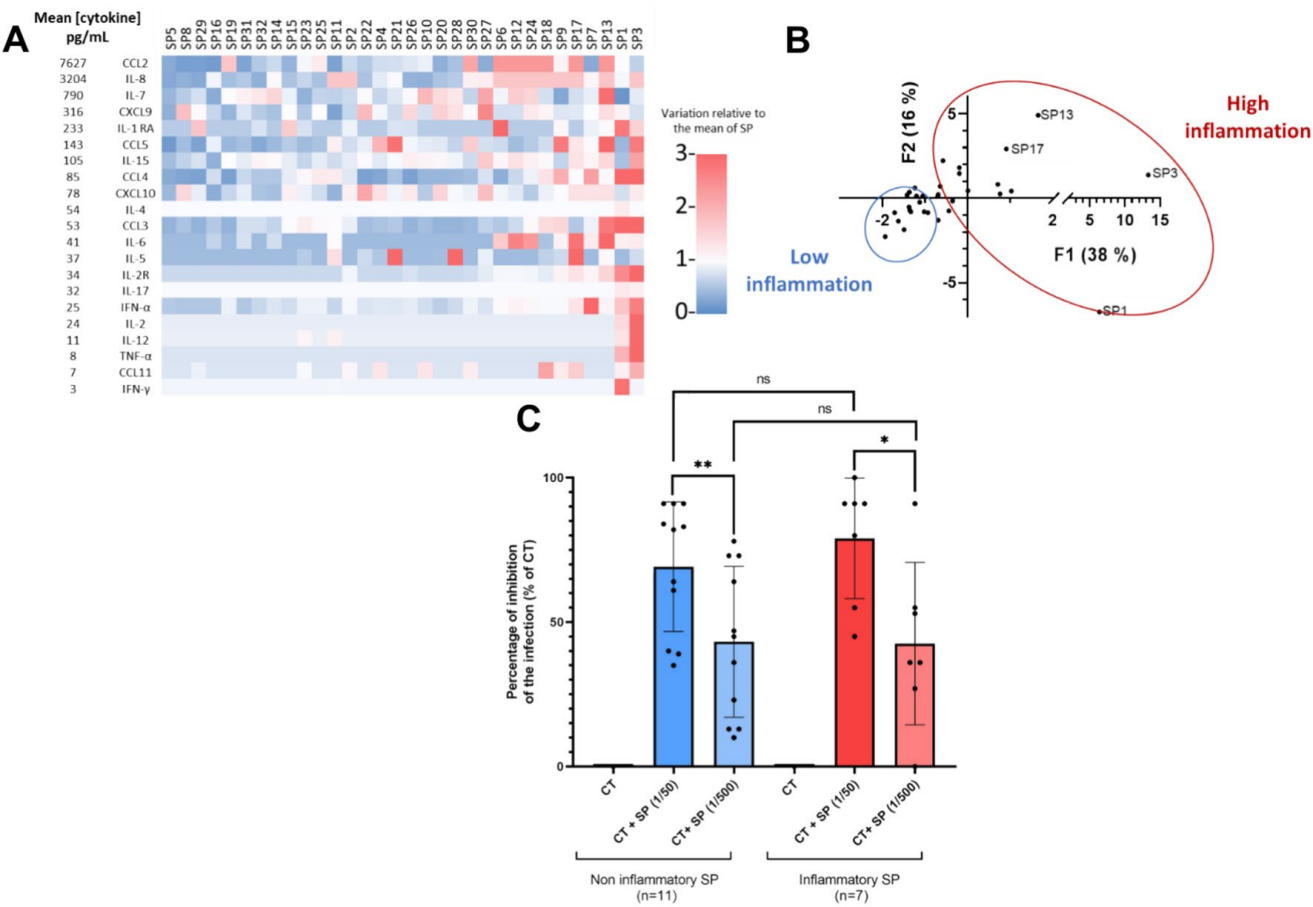
Cytokine concentrations of individual SPs and impact on CT infection of A2EN cells. Cells were infected or not with CT at a MOI of 12, in the presence or not of different dilutions of SP, for 24 h (n = 2). (**A**) Cytokine characterization of the individual SPs. The cytokine concentrations were quantified by Luminex in each of the SP (n = 32). (**B**) A principal component analysis was performed on the cytokine profiles of individual SP, and thus they were classified into two groups, high inflammatory and low inflammatory. (**C**) The percentage of inhibition of CT infection ([percentage of infection in CT condition – percentage of infection in CT + SP condition]/percentage of infection in CT condition) was determined by immunofluorescence at 24hpi by quantification of the inclusion in A2EN infected cells (n = 18). The asterisk indicates a significant difference by T-test (*p ≤ 0.05, **p ≤ 0.01). Figure (**C**) was formulated using GraphPad Prism software.

Given the impact of the pool of SP on CT infection, we also tested the impact of individual SP, isolated from patients with different clinical and inflammatory parameters. The individual SP were characterized in terms of cytokine/chemokine concentrations (Fig. 4A). The individual SP had various inflammatory profiles, we thus classified them into two groups, one “high inflammation”, with more pro-inflammatory cytokines expressed and another “low inflammation” (Fig. 4B). The effect of the individual SP was examined on A2EN cells infected with CT. Several dilutions of SP in PBS were tested. No effect was detected on cell viability. We then determined the percentage of inhibition of CT infection in the various conditions. We showed that for all the individual SP tested, a 50-fold dilution of SP inhibited the percentage of CT infected cells at 24 h post-infection (Fig. 4C). This effect was also dose dependent: the percentage of inhibition decreased for a 500-fold dilution of SP.

### Seminal plasma induced transcriptional changes in A2EN cells

**Fig. 5:**
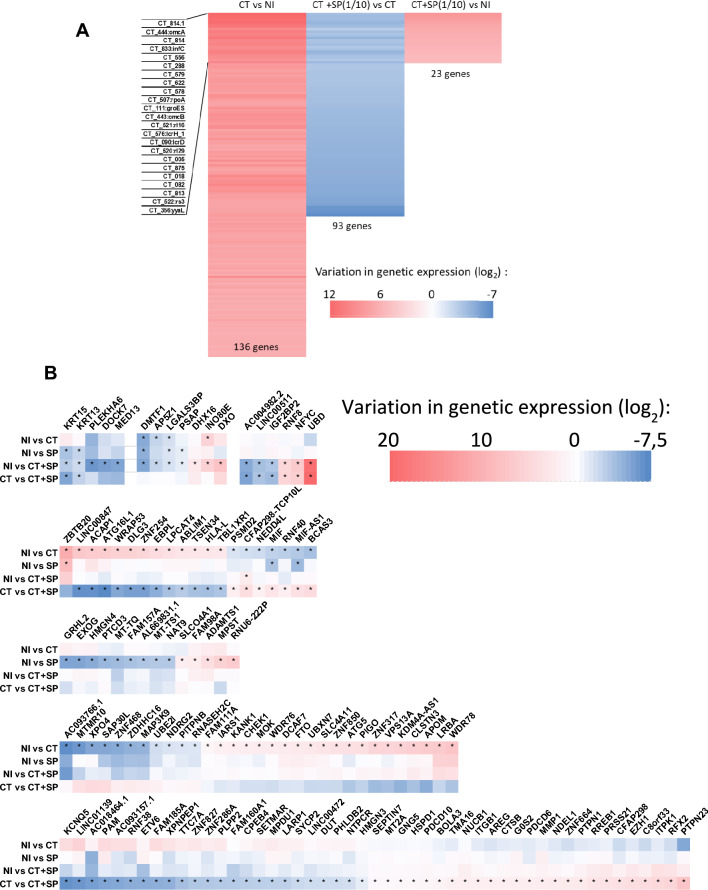
Transcriptomic analysis of A2EN cells infected with CT in presence or not of SP. Cells were infected or not with CT at a MOI of 25 for 48 h, in presence or not of a tenfold dilution of SP and were then lysed for transcriptomic analysis (n = 3). *C. trachomatis* (**A**) or host-responding (**B**) gene expression in A2EN cells exposed to CT, SP or CT + SP. The log_2_ fold change in gene expression was calculated using Enrichr, and is represented compared either to the uninfected/unstimulated (NI) condition or to CT infected cells (*: adjusted p-value < 0.05).

To address the effect on gene expression of the inhibition of CT infection of A2EN cells by the pool of SP, a transcriptomic analysis of A2EN cells in various experimental conditions was performed (Fig. 5A, B). In CT infected A2EN cells, 136 CT genes were detected at 48 h in the transcriptomic profile of A2EN cells. In Fig. 5B, eukaryotic gene groups were assigned based on similarities in their significant genetic variations. In CT infected cells, 55 eukaryotic genes were differentially regulated compared to those in the non-infected (NI): for example, ATG5, ATG16L1, UBE2I, NEDD4L or many ZNF-genes involved in various metabolic processes and in the regulation of gene expression were differentially regulated. During exposition to the pool of SP, genes that were statistically differentially regulated were different from those in NI vs CT infected condition. Some of those genes are metalloproteases (MPST, ADAMTS1). When A2EN cells were infected with CT in presence of the pool of SP, all CT genes were downregulated and only 23 genes were detected at 48 h post-infection compared to the NI condition.

Interestingly, comparing the host related genes, the cells presented a different expression pattern compared to the A2EN cells exposed only to the pool of SP or only to CT. Some genes in the CT vs CT + SP condition were involved in the antigen processing and presentation (NFYC, CTSB). Others are involved in innate immune responses (MIF).

In summary, CT and SP both induce transcriptional changes in A2EN cells. In A2EN cells infected with CT in presence of SP, the transcription profile observed is even different from the one of the non-infected and/or non-SP exposed cells. CT genes are also affected by the presence of SP.

### Impact of A2EN supernatants on neutrophil phenotype and functions

Given the modifications induced by SP ± CT on epithelial cells, we wondered if the variations in the cytokine concentrations could have an impact on the underlying cells in the mucosa. We focused our study on neutrophils since they are the first cells recruited to the site of inflammation. When recruited, neutrophils mostly come from the blood^31^, so we used neutrophils isolated from human blood to assess the impact of A2EN supernatants on their phenotype and functions.

#### Phenotype

**Fig. 6:**
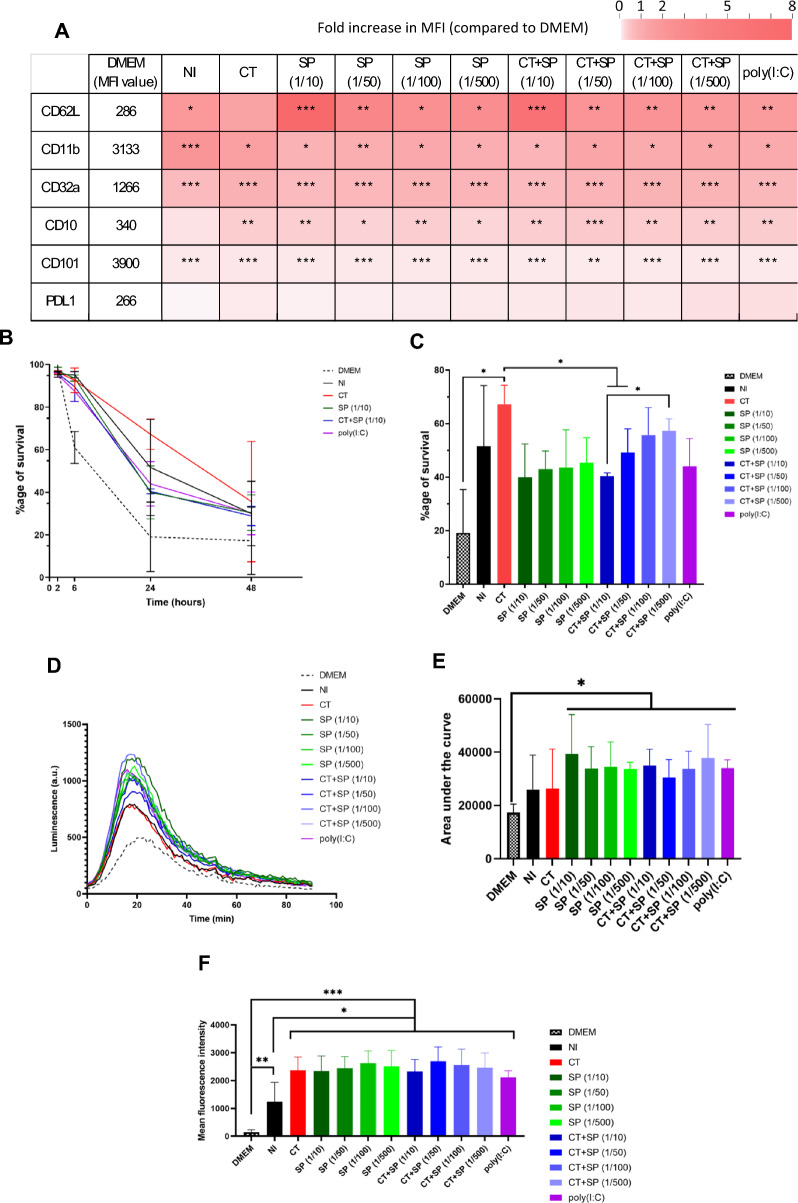
Impact of the A2EN cell supernatants on blood neutrophil. A2EN cells were infected or not with CT at a MOI of 12, in the presence or not of different dilutions of the pool of SP (n = 4). Poly(I:C) was used as a positive control. After 24 h, the supernatants were collected and used on neutrophils isolated from the blood. (**A**) Variation in neutrophil phenotype after 6 h of incubation. The mean fluorescence intensity of CD11b, CD32a, CD10, CD101, CD62L and PD-L1 was compared to DMEM condition on neutrophils (CD45^+^ CD66^+^ Lin (CD3, 8, 14, 20, 123, 125). (**B**) Kinetics of the neutrophil survival after 2 h to 48 h of incubation. Live cells (AnV^−^, 7-AAD^−^) were quantified among neutrophils using FlowJo software, results are expressed as a percentage of survival. (**C**) Variation of neutrophil survival after 24 h of incubation with the different A2EN supernatants. ROS production after 2 h of incubation: PMA was added to stimulated ROS production and luminescence was monitored for 90 min (**D,E**). Area under the curve. (**F**) Phagocytosis assay: after 2 h of incubation, fluorescent *E. coli* particles were added to the neutrophils for 5 min, cells were then fixed and the mean intracellular fluorescence was assessed by flow cytometry. Asterisks indicate a significant difference by two-way ANOVA test (*p ≤ 0.05, **p ≤ 0.01, ***p ≤ 0.001). Figures were formulated using GraphPad Prism software.

First, neutrophil phenotype was analyzed at 0h and 6h post sorting (Supplementary Fig. 4). In DMEM medium, the neutrophil phenotype was comparable between 0 and 6 h post sorting, except for a major decrease in CD62L, and a small decrease in CD101 expression. Then, we assessed the impact of A2EN supernatants on neutrophil phenotype after 6 h of culture. PD-L1 expression on neutrophils was not affected by A2EN supernatants. All supernatants had a quite similar effect on other cellular markers, with an increase in CD11b, CD32a, CD10, CD101 and CD62L expression on neutrophils (Fig. 6A). The A2EN supernatants resulted in a 2.5-fold increase in the mean fluorescence intensity (MFI) of CD11b, CD32a and CD62L, whereas the increase in the MFI of CD10 and CD101 was less important. The decrease in CD62L expression observed in neutrophils after 6 h of culture was less drastic when neutrophils were treated with A2EN supernatants, and more particularly with supernatants from A2EN cells treated with a tenfold dilution of SP. In those conditions, the MFI was at least 7 times more important compared to the DMEM condition. The difference in CD62L MFI was significantly different between the NI and SP (1/10) conditions. In conclusion, in all the tested conditions, A2EN supernatants increased the expression of several surface markers in blood neutrophils.

#### Functions

Neutrophil survival was evaluated from 2 to 48 h post sorting (Fig. 6B). In DMEM medium, the survival of the neutrophils was drastically reduced as soon as 6 h post sorting and continued to diminish at 24 h, with 60% and 20% of cell survival respectively. In contrast, A2EN supernatants enhanced significantly neutrophil survival after 6 h of culture (p < 0.01), with at least 13% and 40% of cell survival at 6 h and 24 h respectively. Supernatants of A2EN cells infected with CT promote neutrophil survival (67.3% of neutrophil survival) compared to the conditions without CT (at best 51.7% of neutrophil survival) (Fig. 6C). SP affected the neutrophil survival in a dose dependant manner. While SP dilution increased, CT infected A2EN supernatants increased the survival of the neutrophils (from 40.6% of neutrophil survival in the 1/10 dilution to 57.5% of neutrophil survival in the 1/500 dilution). Altogether, when treated with SP, the effect of CT infected A2EN supernatants was dependent on the SP dilution: at high SP dilutions, the effect of CT predominated with an enhanced survival of the neutrophils comparing to low dilutions (Fig. 6C).

ROS production was also evaluated by stimulating neutrophils with PMA and monitoring the emission of light by oxidized luminol. A representative graph of the 90 min follow-up is illustrated Fig. 6D. When a PMA stimulation was performed at t = 0 (Fig. 6D, E), neutrophils cultured in DMEM medium were less susceptible to PMA induced ROS production than neutrophils in A2EN treated with SP or poly(I:C) supernatants. Moreover, in DMEM medium, the peak of ROS production occurred a few minutes later than for neutrophils in A2EN supernatants. Without any PMA stimulation, ROS production by neutrophils was very weak and did not differ between the experimental conditions, but neutrophils were still able to produce ROS upon later PMA stimulation (after 50 min in PBS) in all experimental conditions. CT infection did not significantly impact the effect of A2EN supernatants on ROS production. In conclusion, poly(I:C) and SP treated A2EN cell supernatants are potent activators of PMA induced ROS production in neutrophils.

Neutrophil phagocytic capacity was evaluated at 2 h post sorting, at 37 °C and 4 °C. The MFI of neutrophils was evaluated to assess their phagocytic capacity. The MFI for all experimental conditions at 4 °C was lower than at 37 °C. At 37 °C in DMEM, 58% of neutrophils were engaged in the phagocytosis process. The phagocytosis activity was increased in A2EN supernatants, with 83% of neutrophils engaged in phagocytosis. The MFI of the neutrophils in uninfected A2EN supernatants was significantly lower than for the other A2EN supernatants, meaning that their phagocytic activity was significantly increased (Fig. 6F). Indeed, supernatants of stimulated (by poly(I:C), CT and/or SP) A2EN cells caused an increase of at least 70% of neutrophil phagocytic capacity compared to unstimulated A2EN supernatants. In conclusion, A2EN cell supernatants, and more particularly stimulated A2EN cell supernatants are potent activators of phagocytic capacity in neutrophils.

## Discussion

The environment of the FRT, and more particularly the inflammation, is particularly important for the transmission of STI. However, the underlying mechanisms are often poorly or not documented, particularly in the context of co-infections^32^. During this study, we developed an in vitro model aiming at better understanding the role of the SP and the mucosal environment (in terms of inflammation) during CT infection. This model allows to study sequentially the different steps occurring at the level of the FRT in vivo during CT infection: cervicovaginal epithelial cells are infected by CT in presence of SP, then the epithelial cells secrete inflammatory mediators which act on immune cells in the *lamina propria* ^6^.

In vitro and in vivo studies have shown that SP is a potent immunomodulator and affects STI acquisition, either positively or negatively^33^. During male to female transmission of CT, SP acts as a vehicle for the bacteria. In this study, we reported for the first time that SP inhibits CT svD infection of human endocervical epithelial cells. We did not find any correlation between the percentage of CT infection in the different conditions and the cytokine profiles of the individual SP used. The analysis of the transcriptional profile of A2EN cells infected with CT in presence or not of SP revealed that CT genes were more frequently detected in absence of SP. The bacterial genes expressed in the CT and CT + SP conditions were comparable with those found in a previous study of CT infection in HeLa cells^34^. We observed a reduction of many immediate early genes (CT_288, CT_111:groES, CT_529, CT_446:euo, CT_774:cysQ, …) expressed in the CT + SP condition compared to the CT condition. This could suggest a delay in the CT growth in presence of SP, however, we did not find any difference in the size of the inclusion at 24 h post-infection, suggesting an inhibition of early stages of the CT infection, which may include entry stages. The infection inhibition by SP may thus be at several steps: factors in the SP could affect the entry of the bacteria leading to a reduction of the percentage of infection at 24 h and at later stages of the infection, the presence of SP could affect the bacterial growth within the cells at 48 h post-infection. This multi-step model is compatible with the CT life cycle since CT complete its cycle in 48 h to 72 h^35^. Several host genes were also differentially regulated by SP and/or CT. However, we were not able to find general pathways implicated during SP and/or CT exposition. The factor(s) in the SP impacting CT infection do not appear to be the cytokines/chemokines that we have quantified, since all the individual SP tested, with various inflammatory profiles, had the same impact on the percentage of CT infection, suggesting that another factor may be involved. This factor might be another cytokine, that we did not investigate in this study, or defensins for example, that have been described to be present in SP and have been shown to have antimicrobial properties^36^.

A2EN cells infected with CT produce pro-inflammatory cytokines at 24 hpi, but the increase in cytokine concentration was relatively modest compared to poly(I:C) stimulation, as already reported in the literature^5^. For example, IL-8 concentration was not increased by CT or by poly(I:C), or in any other of the experimental conditions. Buckner et al., showed that it was possible to stimulate the production of IL-8 by A2EN cells using poly(I:C), but these results were obtained in different experimental conditions, and with polarized cells. Moreover, the fold increase in the cytokine concentration was correlated with the percentage of CT infection. Also, the basal level of IL-8 was already very high in our experiments, as it has been reported at the level of TRF^37^. This high level of IL-8 has been associated with the recruitment and activation of immune cells^6^.

On the other hand, SP had an anti-inflammatory effect on epithelial cells. The pool of SP we used had a moderate inflammatory profile, and TGB-β was the most concentrated cytokine. This cytokine could be the main modulator of A2EN cytokine production, since it is a known modulator of cytokine production in other cervical cell lines^38^. It has been involved in numerous immunomodulatory processes, notably in accommodating the foreign alloantigens to promote immune tolerance to favor a successful pregnancy^39^. Nevertheless, the anti-inflammatory properties of the SP observed in this study are in contrast to what has been described after a SP inoculation in the FRT mucosa in vivo^39^. This difference can be explained because the in vitro model does not take into account the complexity of the FRT, which contains a diversity of cells (including resident immune cells) that could be responsible for the pro-inflammatory reaction to SP. The cytokines found in the supernatants of A2EN cells infected with CT with SP seem to confirm the anti-inflammatory effect of SP on epithelial cells: the presence of SP leads to modifications in the expression of cytokines by CT-induced A2EN cells. These modifications may be due either to the fact that SP interfere with CT infection cycle, and/or to an additive effect: the expression profile obtained corresponds to the sum of the two profiles obtained with the SP and CT.

We then tested the impact of A2EN supernatants on neutrophils isolated from the blood, since those cells are rapidly recruited after CT infection^6^. However, neutrophils were directly isolated from the peripheral blood, and thus their phenotype may differ from extravasated neutrophils located in vivo at the site of CT infection. Neutrophils, isolated from peripheral blood, were tested for changes in phenotype and function after exposure to A2EN supernatants. The supernatants influenced neutrophil phenotype by increasing levels of CD11b and CD32a, indicating priming, and CD10 and CD101, suggesting maturation ^40^. There was no significant difference according to the A2EN supernatants used. High concentrations of cytokines like IL-8 common in all A2EN supernatants and also found in high concentration at the level of the FRT, may be responsible for this. A2EN supernatants enhance neutrophil survival, likely due to the positive effects of soluble factors within these supernatants. This improvement in survival could primarily be attributed to cytokines like IL-6 and especially GM-CSF, which are significantly reduced in SP-treated A2EN supernatants. GM-CSF is known to affect the expression of neutrophil surface markers^41^, such as reducing CD62L expression in neutrophils and eosinophils ^42,43^. The impact of A2EN supernatants on neutrophil survival corresponded to their cytokine profiles, with varying effects based on the conditions. Cells infected with CT showed the greatest enhancement in neutrophil survival. The cytokines that varied in expression in the supernatants from CT-infected A2EN cells, specifically CCL5, CCL3, CXCL10, IL-6, and GM-CSF, are likely contributors to the observed differences in neutrophil survival.

Both phagocytosis and ROS production were elevated by all A2EN supernatants, potentially driven by cytokines like IL-8, which primes neutrophils for activation^44^ and in conjunction with secondary stimuli like LPS that can trigger phagocytosis and ROS production^45^. The supernatants from non-infected A2EN cells had a lesser impact on phagocytic activity compared to those from infected cells, highlighting the role of infection-driven cytokine environment in enhancing neutrophil functions.

We showed that SP could impact the inflammation at the level of the FRT during CT infection. Those modifications could also impact the risk for STI acquisition and lead to co-infections, so we also tested the impact of A2EN cell supernatants on the susceptibility to HIV-1 infection. However, we were not able to highlight significant differences in U87-CD4-CCR5 susceptibility to a virus pseudotyped with HIV-1 R5 envelope or with VSV-G protein (data not shown). This model allowed to assess the effect of A2EN supernatants in a simplified system with a one cycle viral replication and to evaluate if the supernatant’s impact was occurring at the step of the viral entry. However, it does not recapitulate the full diversity of the immune cells present at the level of the FRT, nor the complexity of a HIV-1 replicative clinical isolate. It will be interesting to test A2EN cell supernatants on primary cells isolated from the FRT, and infected with a HIV-1 R5-tropic virus. Overall, our results highlight the need to take into account SP when studying the mechanisms of STI acquisition. SP has a significant impact on the susceptibility to CT infection and on the innate immune responses to the infection. The interactions between the SP and other factors from the environment at the level of the FRT should be taken into consideration to develop new preventive strategies against STI such as CT.

## Supplementary Information

Supplementary Figures.

## Data Availability

The datasets generated and/or analysed during the current study are available in the NCBI repository, ID 1042933-BioProject-NCBI (nih.gov).
